# A Virtual Community of Practice to Support Physician Uptake of a Novel Abortion Practice: Mixed Methods Case Study

**DOI:** 10.2196/34302

**Published:** 2022-05-05

**Authors:** Sheila Dunn, Sarah Munro, Courtney Devane, Edith Guilbert, Dahn Jeong, Eleni Stroulia, Judith A Soon, Wendy V Norman

**Affiliations:** 1 Department of Family and Community Medicine University of Toronto Toronto, ON Canada; 2 Women's College Research Institute Women's College Hospital Toronto, ON Canada; 3 Department of Obstetrics and Gynaecology University of British Columbia Vancouver, BC Canada; 4 Centre for Health Evaluation and Outcome Science University of British Columbia Vancouver, BC Canada; 5 School of Nursing Faculty of Applied Science University of British Columbia Vancouver, BC Canada; 6 Department of Obstetrics, Gynecology and Reproduction Laval University Quebec City, QC Canada; 7 School of Population and Public Health Faculty of Medicine University of British Columbia Vancouver, BC Canada; 8 Department of Computer Science Faculty of Science University of Alberta Edmonton, AB Canada; 9 Contraception and Abortion Research Team Women's Health Research Institute Provincial Health Services Authority Vancouver, BC Canada; 10 Department of Family Practice University of British Columbia Vancouver, BC Canada; 11 Faculty of Public Health & Policy London School of Hygiene & Tropical Medicine London United Kingdom

**Keywords:** mifepristone, abortion, community of practice, virtual community of practice, diffusion of innovation, learning community

## Abstract

**Background:**

Virtual communities of practice (VCoPs) have been used to support innovation and quality in clinical care. The drug mifepristone was introduced in Canada in 2017 for medical abortion. We created a VCoP to support implementation of mifepristone abortion practice across Canada.

**Objective:**

The aim of this study was to describe the development and use of the Canadian Abortion Providers Support-Communauté de pratique canadienne sur l’avortement (CAPS-CPCA) VCoP and explore physicians’ experience with CAPS-CPCA and their views on its value in supporting implementation.

**Methods:**

This was a mixed methods intrinsic case study of Canadian health care providers’ use and physicians’ perceptions of the CAPS-CPCA VCoP during the first 2 years of a novel practice. We sampled both physicians who joined the CAPS-CPCA VCoP and those who were interested in providing the novel practice but did not join the VCoP. We designed the VCoP features to address known and discovered barriers to implementation of medication abortion in primary care. Our secure web-based platform allowed asynchronous access to information, practice resources, clinical support, discussion forums, and email notices. We collected data from the platform and through surveys of physician members as well as interviews with physician members and nonmembers. We analyzed descriptive statistics for website metrics, physicians’ characteristics and practices, and their use of the VCoP. We used qualitative methods to explore the physicians’ experiences and perceptions of the VCoP.

**Results:**

From January 1, 2017, to June 30, 2019, a total of 430 physicians representing all provinces and territories in Canada joined the VCoP and 222 (51.6%) completed a baseline survey. Of these 222 respondents, 156 (70.3%) were family physicians, 170 (80.2%) were women, and 78 (35.1%) had no prior abortion experience. In a survey conducted 12 months after baseline, 77.9% (120/154) of the respondents stated that they had provided mifepristone abortion and 33.9% (43/127) said the VCoP had been important or very important. Logging in to the site was burdensome for some, but members valued downloadable resources such as patient information sheets, consent forms, and clinical checklists. They found email announcements helpful for keeping up to date with changing regulations. Few asked clinical questions to the VCoP experts, but physicians felt that this feature was important for isolated or rural providers. Information collected through member polls about health system barriers to implementation was used in the project’s knowledge translation activities with policy makers to mitigate these barriers.

**Conclusions:**

A VCoP developed to address known and discovered barriers to uptake of a novel medication abortion method engaged physicians from across Canada and supported some, including those with no prior abortion experience, to implement this practice.

**International Registered Report Identifier (IRRID):**

RR2-10.1136/bmjopen-2018-028443

## Introduction

### Background

Communities of practice (CoPs) are recognized as tools for enhancing knowledge, improving practice, and supporting innovation [1,2]. As described by Wenger et al [3], CoPs are “groups of people who share a concern, a set of problems or a passion about a topic and who deepen their knowledge and expertise in this area by interacting on an ongoing basis.” In health care, CoPs have been used to exchange information and knowledge, support implementation of practice innovations, build a sense of identity, and reduce professional isolation [1,4-6]. Virtual CoPs (VCoPs) can achieve these goals among geographically dispersed practitioners [5,7]. We hypothesized that a VCoP could be particularly valuable to promote the adoption of a novel medical practice (mifepristone abortion care) introduced in Canada in 2017 and to facilitate uptake of this practice by primary care providers, particularly those in rural and remote regions who may have limited professional support and resources [7-9].

Mifepristone, when used in combination with misoprostol, is recognized internationally as the *gold standard* for medication abortion [10]. Since its first approval in France in 1988, mifepristone has been approved in more than 79 countries and has been used by millions of people worldwide [11]. Mifepristone is safe, effective, and as a straightforward alternative to surgical abortion has transformed the way abortion is provided; its introduction in Canada in 2017 raised the important question of how health care professionals could be helped to implement this innovation.

In preparation for mifepristone’s availability in 2017, the Contraception and Abortion Research Team-Groupe de recherche sur l’avortement et la contraception [12] launched the CART-Mife Study, a 4-year national implementation research project, described elsewhere [13], that aimed to identify and mitigate barriers to implementing mifepristone abortion practice, particularly those affecting community-based physicians and pharmacists. The project had two interventions: (1) integrated knowledge translation with health policy makers to mitigate health system barriers and support facilitators to adoption of mifepristone abortion practice by physicians and pharmacists and (2) a VCoP [14], which was established to address the needs of community-based physicians and pharmacists across Canada who were interested in adopting mifepristone abortion practice in their professional roles.

Canada’s laws, regulations, geography, and health system present challenges and opportunities for mifepristone abortion practice that are distinct from those in other countries. Almost unique in the world, Canada has no criminal law on abortion [15]. Since 1988, abortion has been considered a medical procedure and its need determined by the patient with their health care provider [16]. In addition to imposing varied criminal sanctions on abortion, most high-income countries also highly regulate how mifepristone is prescribed and dispensed, as well as where it is used [17-19]. Mifepristone’s initial Canadian approval in 2015 had several similar regulatory restrictions, most of which were removed over the 2 years after its availability in 2017 [20,21]. Currently, many countries restrict mifepristone provision to certain types of practitioners, such as medical specialists or registered approved providers, or purpose-specific facilities [22-24]. Except for the province of Quebec [25], Canada has eliminated such restrictions and allows prescription by any authorized prescriber (physicians and, in most provinces, also nurse practitioners) [26]. Some countries, including the United States, have not allowed pharmacies to dispense mifepristone but require drug dispensing by the prescriber or clinic [17,24]. Not so in Canada; by November 2017, mifepristone became available from pharmacies like any other drug, to be dispensed by any pharmacist when presented with a prescription [26]. Government health insurance plans cover costs of the drug. The requirement for preabortion ultrasound was removed in April 2019 [27]. Although ultrasound is often used, clinical guidelines and mifepristone drug approvals in most other countries, including the United States, do not require it [10,17,19]. Canada’s lack of restrictions opened the door for mifepristone abortion provision in community primary care. This globally unique situation presented an opportunity to address the considerable geographical challenges to abortion access faced by Canadians living in rural or remote communities, distant from the large metropolitan centers where most abortion services are located [28]. Availability in community primary care could provide patients with local access to abortion through their own health care provider.

To promote widespread uptake of mifepristone abortion practice, addressing nonregulatory barriers to implementation was also crucial. Medication abortion care is not complicated or difficult, but in 2017 few Canadian physicians were knowledgeable about, or had experience with, it. Abortion is also highly stigmatized. The fear of negative attitudes or harassment from colleagues, patients, and local communities could inhibit and isolate abortion providers [29-32]. In 2017, Dawson et al [33] identified challenges and facilitators experienced by primary care medication abortion providers in Australia, whose publicly funded health system and mifepristone drug approval are similar to those in Canada. Barriers included not recognizing medication abortion as within the physician’s scope of practice; stigma; logistical challenges such as finding a pharmacy with the drug, access to ultrasound, consent forms, and patient information sheets; lack of experience, access to experts, mentorship, and peer support; and professional isolation. In Canada, Dressler et al [8] found that rural physicians also experienced professional isolation and lack of training opportunities.

We theorized that information, resources, tools for practice, and an accessible professional network to access and share implementation enablers would enhance the ability, and perhaps willingness, of approved health care providers (initially only physicians and pharmacists) to provide medication abortion care. Furthermore, we theorized that real-time collection of reported barriers could inform health system and regulatory decision-makers’ understanding and ability to address unanticipated barriers. Working with national health professional organizations, guideline committees, and government regulators, we developed a national VCoP with these features. Our VCoP went live in January 2017, at the same time that mifepristone became commercially available.

### Objectives

This paper describes the development of the Canadian Abortion Providers Support-Communauté de pratique Canadienne sur l’avortement (CAPS-CPCA) VCoP, examines its use, and explores the perspectives of physicians, who were the only eligible prescribers at the start of the study period, on its value for implementing this novel clinical practice.

## Methods

We adopted an intrinsic case study approach using mixed methods during the study period January 1, 2017, to October 30, 2019. Intrinsic case studies are used to explore a specific event or issue in depth in a real-life context [34].

### Theoretical Framework for CAPS-CPCA

**Figure 1:**
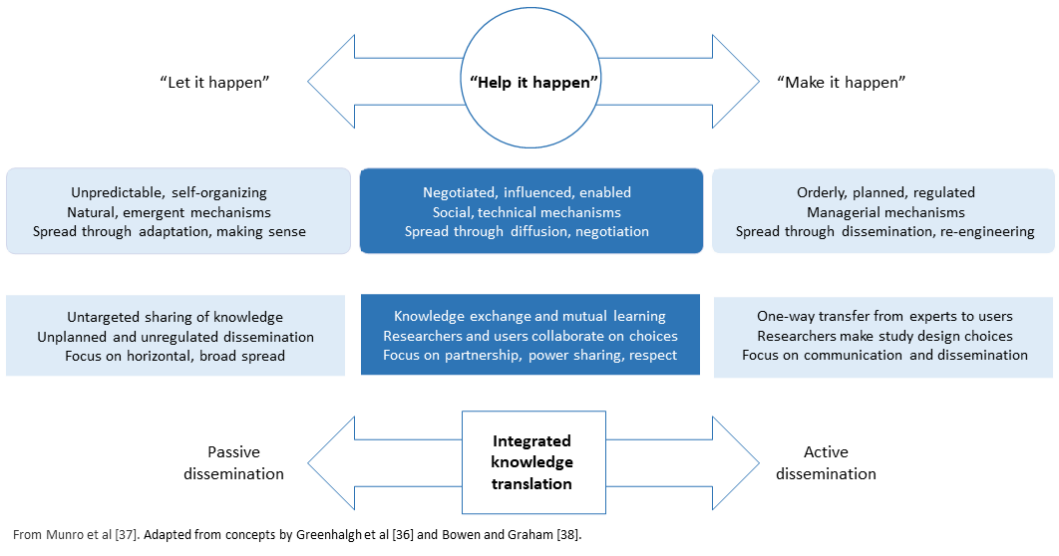
Conceptual framework for diffusion of innovation (reproduced from Munro et al [37], which is published under Creative Commons Attribution 4.0 International License [39].

Our development of CAPS-CPCA was informed by the *Theory of the Diffusion of Innovation* formulated by Rogers [35] as operationalized by Greenhalgh et al [36]. Greenhalgh et al [36] theorized that the implementation of innovations in health systems is affected by a complex interaction of influences. These include characteristics of the innovation (complexity, compatibility, advantage, trialability, and observability) and the adopter (motivation, skills, and values), system readiness for the innovation (tension for change, innovation-system fit, and dedicated resources), mechanisms used for implementation (technical support and social networks), the outer context of regulatory and sociopolitical influences, and the communication and influence of change agents and knowledge purveyors. We conceptualized the CAPS-CPCA VCoP as a mechanism to support this innovation in abortion practice—in the words of Greenhalgh et al [36], to “Help it happen”—through both social and technical means [37] (Figure 1 [36-38]).

We anticipated that most VCoP members would share common attitudes (homophily) and be motivated to join because of an interest in, and commitment to, women’s reproductive health that included abortion. The VCoP features aimed to decrease complexity and increase compatibility of the innovation, explain and improve the innovation’s relative advantage for adopters, reduce its perceived risks, and create a social network to enhance knowledge and share experience and expertise. We also used the CAPS-CPCA VCoP as a tool to identify physician and pharmacist experiences of health policy and systems barriers to implementation—findings that informed the main project’s integrated knowledge translation activities with health policy decision-makers to mitigate or eliminate these barriers (the regulatory and sociopolitical influences described by Greenhalgh et al [36]), and further help these practitioners to adopt this practice [13,37]. Integrated knowledge translation is the process of including key stakeholders in all stages of the research process, which in our study included discussing the barriers and facilitators data collected through the CAPS-CPCA VCoP with federal decision-makers in real time.

### CoP Development

The CAPS-CPCA web-based platform (Multimedia Appendix 1) allowed members asynchronous access through their personal account to site content (in English and French), to find pharmacies in their community dispensing mifepristone, to post comments and tips, or ask questions. CAPS-CPCA aimed to encourage multidirectional interaction among members, experts, and researchers to promote sharing information of best practice resources and practice facilitators. Its features specifically addressed barriers to medication abortion practice that had been identified in the literature or were identified during the research project [8,33] (Table 1).

**Table 1:** Features of the virtual community of practice addressing barriers and facilitators to mifepristone abortion uptake and related Diffusion of Innovation constructs.

Barrier or facilitator (Diffusion of Innovation constructs addressed)	CAPS-CPCA^a^ feature
Lack of clinical knowledge (advantage, complexity, experience, risk, and observability)	Clinical practice guidelines and reviews Frequently Asked Questions Email Member Announcements
Tools for practice (complexity and compatibility)	Sample forms (consent, patient information, and follow-up forms, as well as charting forms and checklists) Patient resources Billing codes
Logistical challenges (social values, trialability, diffusion and influence, and system readiness)	Discussion Room Map of pharmacies stocking mifepristone “What’s happening in your province?” Member polls
Peer support and access to experts (social values, trialability, diffusion and influence, and system readiness)	Discussion Room Ask an Expert
Isolation and stigma (social values, trialability, diffusion and influence, and system readiness)	Membership in CAPS-CPCA Discussion Room Ask an Expert Email Member Announcements Member polls

^a^CAPS-CPCA: Canadian Abortion Providers Support-Communauté de pratique canadienne sur l’avortement.

To address the desire for peer support, expert advice, and mentorship, CAPS-CPCA had participative *Discussion Room* and *Ask an Expert* features. Members could pose clinical or practice-related questions and receive a response from an experienced Canadian abortion provider within 24 hours. To maintain confidentiality, questions and answers were not directly visible to all site users and all posted interactions were identified only by the user's ID: a random number tagged with a professional 2-letter designation (eg, 3174MD and 2061NP). Reoccurring clinical questions were rephrased and shared with all users through *Member Announcements* and *Frequently Asked Questions* features.

We sought formal support from professional organizations representing most of the anticipated providers from across Canada (ie, family medicine, obstetrics and gynecology, nursing, and pharmacy) for their social influence among their members to confirm that mifepristone abortion was within their members’ scope of practice and build credibility of the VCoP. We included their organizational logos in branding materials. Research team members were recognized local and national experts in their disciplines and promoted CAPS-CPCA through their professional networks. To further inspire confidence and minimize perceived conflict of interest, we received no industry funding and did not disseminate industry-prepared materials.

Throughout the project we responded to member requests for additional support; for example, we created resources such as clinical checklists and guideline summaries [40]. Acting on early feedback that many members preferred email notifications rather than logging in to the website, we began emailing *Member Announcements* containing brief information on policy updates, continuing education events, common practice questions, relevant research, and product shortages. Finally, email polls allowed members to contribute information about the real-time impact of health policies, such as the early requirement for prescribers to register with the manufacturer or for a mandatory ultrasound to initiate medication abortion, knowing that the member’s perspective would be used to inform policy decisions.

### CAPS-CPCA Member Recruitment

Vigorous recruitment was a key strategy for community building to reduce isolation and stigma associated with abortion practice. We invited interested physicians and pharmacists from across Canada to join CAPS-CPCA. This recruitment occurred primarily through a web-based medical abortion training course hosted by the Society of Obstetricians and Gynaecologists of Canada that, until November 2017, was a prerequisite to prescribe or dispense mifepristone [41]. Other recruitment occurred through continuing education events, word of mouth, announcements from our partner organizations to their members, at the federal drug regulator’s drug information site and in its communications distributed to all practicing physicians, and on the product website. After the removal of Health Canada’s regulation for physician-only prescribing in November 2017, CAPS-CPCA extended its membership to nurse practitioners and midwives through their professional organizations.

Acknowledging concerns about safety and potential for harassment, membership was restricted to licensed health professionals (physicians, pharmacists, and later nurse practitioners and midwives), their verified staff, health professional trainees, and project collaborators. Internal firewalls permitted only the licensed health professionals to access the site’s clinical discussion and expert advice areas. Membership requests were made on the web and vetted by the research team by verifying the applicant’s professional license and requesting references, if needed.

### Data Sources

We collected data from three sources during the study period:

1. CAPS-CPCA website data and *Member Announcements* WordPress data collected from January 1, 2017, to June 30, 2019, included member details, page views or downloads accessible only to members, resource views or downloads accessible from the landing page, *Member Announcements* emails opened, and email poll responses. We also collected content from *Ask an Expert* questions and *Discussion Room* threads with physician posts.
2. Electronic surveys were completed as part of the main CART-Mife Study by CAPS-CPCA physician members and nonmembers who were interested in providing medication abortion. Survey development is described elsewhere [42]. Surveys were administered in English or French at baseline, 6 months, and 12 months (last 12-month survey collected in October 2019) to collect data on clinician characteristics and practices as well as barriers and facilitators to implementation (Multimedia Appendices 2 and 3). Follow-up surveys included 7 questions about CAPS-CPCA participation, its importance, and suggestions for improvement.
3. Interviews: As part of the main study, we conducted semistructured interviews in English or French with a national sample of abortion-providing and nonproviding physicians, including a subset of survey respondents, as well as health system stakeholders. Details of the qualitative study design and results of the interviews are reported elsewhere [13,21,25,37,43]. Interviews were conducted by telephone in English or French by a knowledge translation scientist (SM), physician researcher (EG), and nursing doctoral student (CD). The interview questions probed for domains of the Diffusion of Innovation theory. Of relevance to CAPS-CPCA, specific questions explored VCoP membership, how it did or did not support prescribers, and their experience and overall thoughts about the VCoP. For participants who had not accessed CAPS-CPCA, we asked if joining this website would be useful (why or why not), what information they would want from the website, and what features they liked about other CoPs.

Although all CAPS-CPCA members representing diverse health professions (physicians, pharmacists, nurses and nurse practitioners, and midwives) are included in the overall site use data, the data from surveys, interviews, and website *Ask an Expert* and *Discussion Room* content used in this analysis relate only to physicians, who were the only health care providers initially eligible to prescribe mifepristone and who made up most (430/521, 82.5%) of the eligible prescribers throughout the study.

### Data Analysis

Site metrics, CAPS-CPCA member characteristics, and responses to survey questions were analyzed descriptively (counts, means, medians, and percentages), and we used chi-square statistics to examine the association of member characteristics with members’ reported use and perceived importance of the VCoP with significance set at *P*<.05. Website page views were aggregated from the webserver logs using AWStats [44]. We analyzed qualitative data (website threads, open-ended survey responses, and interviews) drawing from directed content analysis and thematic analysis approaches, using concepts from the Diffusion of Innovation theory as guiding deductive codes, which we then tested and refined with inductive coding [35,45-47]. We examined and categorized content from *Ask an Expert* and *Discussion* *Room* threads as related to system and regulatory, implementation and logistical, or clinical issues. We analyzed audio-recorded, transcribed interviews for themes related to our key objectives for this substudy and explored physician participants’ use of the CAPS-CPCA and their perspectives on the value of the VCoP for implementing this clinical practice. Methods of thematic analysis and additional results of our analysis of the interviews are described in previous publications [21,25,43]. Using mixed methods techniques, we triangulated our data concurrently with individual data set analyses to compare and contrast findings and gain a deeper understanding of how members used the CAPS-CPCA VCoP and why [48].

### Ethics Approval

The CART-Mife Study received ethics approval from the Behavioural Research Ethics Board at the University of British Columbia (H16-01006).

## Results

### Overview

**Figure 2:**
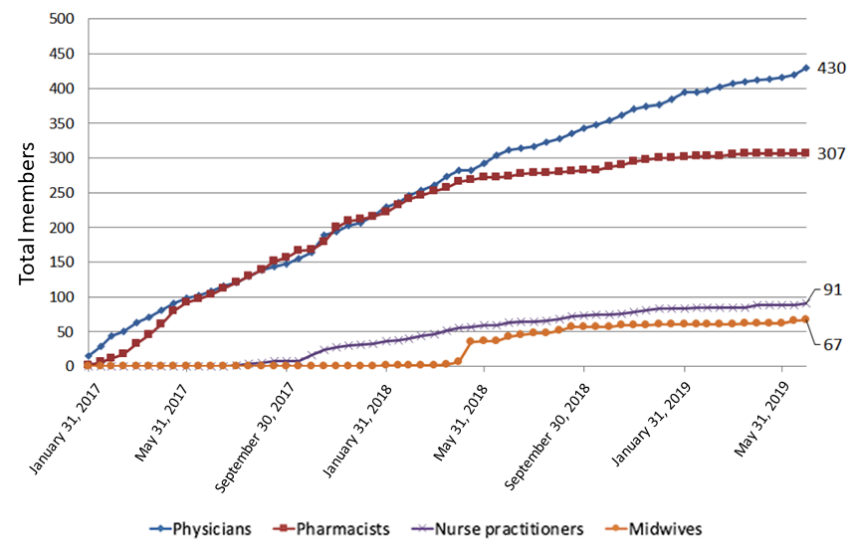
Canadian Abortion Providers Support-Communauté de pratique canadienne sur l’avortement clinician membership over time.

Over the first 30 months, CAPS-CPCA membership grew steadily, accepting more than 1000 members representing all provinces and territories, including 430 physicians (Figure 2). Of the 430 CAPS-CPCA physicians, 222 (51.6%) participated in the baseline survey available from January 2017 until April 2019, which collected demographics and abortion experience (Table 2). Of the 222 respondents, 170 (80.2%) were female; 156 (70.3%) were family physicians; 15 (6.8%) practiced in regions with no abortion services before January 2017; 78 (35.1%) had no previous abortion experience; and, notably, 123 (55.4%) practiced outside metropolitan areas, although only 29.5% of the Canadian population live there [49].

**Table 2:** CAPS-CPCA^a^ virtual community of practice: characteristics of physician members who completed a baseline survey (N=222).

Characteristic			Values, n (%)
**Sex**			
	Female	170 (80.2)	
	Male	38 (17.1)	
	Other or missing	6 (2.7)	
**Age (years)**			
	<35	60 (27)	
	35 to 44	56 (25.2)	
	45 to 54	53 (23.9)	
	>54	47 (21.2)	
	Missing	6 (2.7)	
**Primary specialty**			
	Family or general practice	156 (70.3)	
	Obstetrician-gynecologist	53 (23.9)	
	Medical student or resident	10 (4.5)	
	Other or missing	3 (1.4)	
**Province (% of Canadian population)** **[50]**			
	Ontario (38.6)	82 (36.9)	
	British Columbia (13.5)	39 (17.6)	
	Quebec (22.6)	30 (13.5)	
	Nova Scotia (2.6)	17 (7.7)	
	Saskatchewan (3.1)	13 (5.9)	
	Alberta (11.6)	12 (5.4)	
	Manitoba (3.6)	6 (2.7)	
	Atlantic provinces^b^ (3.9)	10 (4.5)	
	Northern territories^c^ (0.3)	9 (4.1)	
	Missing	4 (1.8)	
**Residence location** **(% of Canadian population)** **[49]**			
	Large metropolitan area (71.8)	94 (42.3)	
	Outside large metropolitan area (29.5)	123 (55.4)	
	Missing	5 (2.3)	
**Previous abortion experience**			
	None	78 (35.1)	
	Medical and surgical	71 (32)	
	Medical only	36 (16.2)	
	Surgical only	33 (14.9)	
	Missing	4 (1.8)	
**Primary facility type**			
	Private physician office	78 (35.2)	
	Community abortion or reproductive health clinic	40 (18)	
	General health care community or ambulatory clinic	25 (11.3)	
	Hospital-affiliated facility	54 (24.3)	
	Other	5 (2.3)	
	Missing	20 (9)	
**Other abortion services available in the community**			
	Medical and surgical	134 (60.4)	
	Surgical only	33 (14.9)	
	Medical only	20 (9)	
	None	15 (6.8)	
	Missing	20 (9)	
**Do you currently, or do you plan to, prescribe mifepristone?**			
	Yes	144 (64.9)	
	No	16 (7.2)	
	Missing	62 (27.9)	

^a^CAPS-CPCA: Canadian Abortion Providers Support-Communauté de pratique canadienne sur l’avortement.

^b^New Brunswick, Newfoundland and Labrador, and Prince Edward Island were combined because of small cell sizes.

^c^Yukon, Northwest Territories, and Nunavut were combined because of small cell sizes.

### Website Data

**Figure 3:**
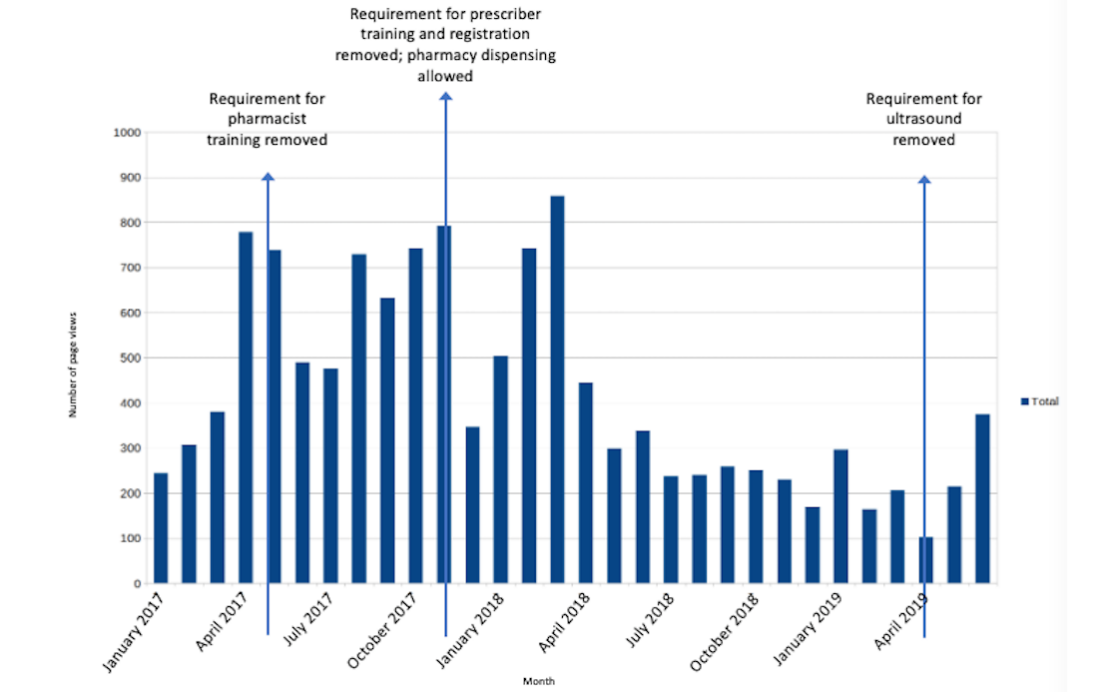
Page views from January 1, 2017, to June 30, 2019, with regulatory-change dates.

Website traffic is shown in Figure 3. Traffic peaked in the first half of the study period and then declined but remained stable. The most frequently visited pages were *Helpful Resources* (2338/12,592, 18.57%, page visits), *Locate a Pharmacy* (2154/12,592, 17.11%), *Ask an Expert* (1792/12,592, 14.23%), and *Latest News* (1892/12,592, 15.03%). From January 1, 2017, to June 30, 2019, there were more than 10,000 views or downloads of resources, including some (ie, *Prescriber* and *Pharmacist Checklists* and *Prescriber* and *Pharmacist Resource Guides*) which, at the request of Health Canada, were made openly available on the CAPS-CPCA landing page and thus were not exclusive to members. The *Prescriber Resource Guide* and *Prescriber Checklist* were viewed or downloaded 1759 times. Other resources accessible only to VCoP members and most relevant to prescribers, such as consent forms, patient information sheets, pharmacy locations, and information on coverage for drug costs across the country, had 1263 views or downloads.

Each of the 77 email *Member Announcements* was opened an average of 341.8 (SD 73.3) times. Response to 2 email polls distributed on May 30, 2017, and March 22, 2019, was 48% (47/99) of the physicians and 5.7% (28/489) of the prescribers (physicians and nurse practitioners), respectively. The first poll asked about the early requirement for physicians to register with the pharmaceutical company to prescribe and dispense mifepristone, similar to a current requirement in the United States [17]. The respondents did not support this restrictive requirement, which also violated codes of conduct of some provincial licensing bodies [51]. It was removed the same week that the poll results were shared with the regulatory decision-makers. The second poll asked about the requirement for pelvic ultrasound in the initial drug approval [20]. Of the 28 respondents, 9 (32%) said that mandatory ultrasound limited their ability to provide mifepristone abortion. Health Canada subsequently removed this requirement [27].

During the 30-month website data collection period, physicians posed 38 *Ask an Expert* questions and there were 19 physician posts in 14 *Discussion Room* threads. Of the 52 questions and discussions, 12 (23%) related to health system or regulatory issues such as how to access mifepristone through pharmacies and hospitals, access to surgical abortion for failed abortions, billing for medication abortion, and drug shortages; 12 (23%) related to logistics of implementation, such as on-call coverage, considerations in rural and remote areas, and overcoming resistance of colleagues or hospitals to mifepristone abortion; and 28 (54%) were clinical questions ranging from use of mifepristone in specific circumstances (eg, breastfeeding, breast cancer, and opiate user), more complicated clinical courses (eg, lack of bleeding or persistent bleeding), and recommended practice when clinical or laboratory resources were limited (eg, access to ultrasound and management of Rh-negative patients).

### Survey Data

CAPS-CPCA members constituted 56.3% (129/229) of the respondents in the 6-month physician follow-up survey, with 66.7% (86/129) of these member respondents agreeing or strongly agreeing that the VCoP was helpful (data not shown). The 12-month follow-up surveys were completed by 224 physicians, of whom 127 (56.7%) indicated that they were still CAPS-CPCA members (Table 3). Of these 127 members, 81 (63.8%) said that the availability of a web-based support platform had been important or very important to them when deciding to provide mifepristone. Perceived importance of the VCoP was not associated with physician experience or urban or rural location. At 12 months, 59.1% (75/127) of the physicians intended to remain members of CAPS-CPCA. In open-ended responses, many members commented that logging in to the site was burdensome and that site navigation should be improved but they valued email updates and the resources and tools for practice. Several remarked that for clinical concerns, they preferred local professional contacts rather than CAPS-CPCA peers or experts because “CAPS...is less helpful for in-the-moment clinical support.” Others recommended that CAPS-CPCA build a centralized resource to support referrals for needed clinical backup and a larger list of pharmacies stocking the medication. Of the 57 respondents who said that they were not members, 34 (58%) had not heard of CAPS-CPCA and those who *were* aware of the VCoP cited no need for it and lack of time as the main reasons for not joining.

**Table 3:** Members’ opinions about Canadian Abortion Providers Support-Communauté de pratique canadienne sur l’avortement (CAPS-CPCA): 12-month–survey responses.

		Values, n (%)
**Respondents to 12-month survey (N=224)**		
	CAPS-CPCA members	127 (56.7)
	Nonmembers	57 (25.4)
	Missing	40 (17.9)
**How important was it to know there was an online platform for support when you decided to provide mifepristone? (n=127)^a^**		
	Important or very important	81 (63.8)
	Neutral or not important	46 (36.2)
**Now (after 1 year), how important has the CoP^b^ been? (n=127)^a^**		
	Important or very important	43 (33.8)
	Neutral or not important	70 (55.1)
	Missing	14 (11)
**Do you plan to continue to participate in CAPS-CPCA? (n=127)^a^**		
	Yes	75 (59.1)
	No	13 (10.2)
	Don’t know	39 (30.7)
**How many times in the past 12 months have you accessed CAPS-CPCA? (n=127)^a^**		
	0	34 (26.8)
	1 to 2	44 (34.6)
	3 to 5	25 (19.7)
	>5	18 (14.2)
	Missing	6 (4.7)

^a^Only those stating that they were members of Canadian Abortion Providers Support-Communauté de pratique canadienne sur l’avortement were asked questions related to the virtual community of practice.

^b^COP: community of practice.

By 12 months, 77.9% (120/154) of the respondents who had ever been a CAPS-CPCA member, including 65% (34/52) with no previous abortion experience, indicated that they had provided mifepristone abortions (median 20, IQR 3-50).

### Interview Data

Over the first year of CAPS-CPCA, we conducted interviews with 55 physicians as part of our broader study on implementation of mifepristone in Canada [13,21], including 33 (60%) members and 22 (40%) nonmembers, 91% (20/22) of whom had not heard of CAPS-CPCA. Analysis identified the following key themes about CAPS-CPCA among members: sense of community and support, clinical usefulness of practice tools, the importance of access to clinical support, importance of CAPS-CPCA for keeping up to date on regulatory changes, preference for emails for information, and concerns about security (Textbox 1). Some interviewees were unfamiliar, or had not engaged, with CAPS-CPCA but felt that access to experts, practice tools, and information on regulations would be valuable in a VCoP.

**Thematic analysis of physician interviews.**

**Sense of community and support**

- Canadian Abortion Providers Support-Communauté de pratique canadienne sur l’avortement (CAPS-CPCA) gave members a sense of community and support, often described as a sense of a community of practitioners, spread nationwide. A participant stated, “Well, it’s just that you don’t feel so alone” [Physician 013], and another said, “We need to have platforms that bring providers together to deal with whatever issues are arising” [Physician 018]. Another commented, “I also sort of just like the feeling that it makes you part of a community with people with common interests” [Physician 015]. Some felt that it was especially important for new providers and could increase their confidence to adopt the practice: “If you were a new provider going to it you would get the guidelines...the checklists...support, if you needed it” [Physician 002]; “A great resource to even tell people that are thinking about doing this and are feeling a little less confident” [Physician 019].

**Clinical usefulness of practice tools**

- Members and nonmembers agreed about the usefulness of downloadable practice support tools such as consent forms, guidelines, and checklists, as well as a more extensive list of dispensing pharmacies. Participants who acted as informal mentors described CAPS-CPCA as their go-to resource for educating new mifepristone providers and linking them with practice tools. In turn, participants provided suggestions on useful practice tools, which informed how we organized and shared resources on the platform: “If anything, I would say have more handouts that you could print off and give to patients...it would be nice to go to that abortion providers’ website and just go, ‘I know where I can find it,’ because exactly. Sometimes, you’re scrambling” [Physician 033].

**Importance of clinical support**

- Members and nonmembers articulated a need for access to clinical support. Some had established and preferred local contacts: “I would feel more comfortable just phoning up the obstetricians I have a close relationship with than to post something on a board and have people that I don’t have [a] relationship with answer” [Physician 025]. However, they also felt that “[CAPS-CPCA is] an excellent resource and community...[to] go get support” [Physician 006], particularly for solo or rural physicians without a local support network: “I think for people that may be more kind of solo or in a group of family doctors, that may be a really helpful place to ask opinions on kind of situations you might come across” [Physician 004]. A participant expressed a desire for personalized mentorship: “It would also be good to have almost like a mentor to just touch base with every once in a while, potentially also to discuss more difficult cases” [Physician 042], and another wanted more local or regional subgroups for direct communication among members.

**Importance of CAPS-CPCA for keeping up to date with information and regulatory changes**

- “I’ve no idea how I would learn about [changing regulations] though, if I weren’t on CAPS-CPCA” [Physician 016]. In early interviews, CAPS-CPCA members indicated a preference for emails for information and “tend[ed] to go less on the website” [Physician 002] because logging in was burdensome. However, some found the emails too frequent and even “intrusive” [Physician 013].

**Concerns about security**

- Physicians accepted that security issues were part of abortion care and stressed the importance of website security in that “people are pretty cautious about sort of publicly being identified as abortion providers” [Physician 035]. Members appreciated the fact that the website was run by known leaders as well as the process for member authentication. However, concerns about “databases where my name and info are potentially breachable” kept a physician from joining [Physician 035]. Physicians were skeptical about pharmaceutical industry involvement in clinical practice and a member voiced concern that CAPS-CPCA might be a “vehicle for promotion” for pharmaceutical companies [Physician 013].

**Lack of awareness and engagement with the virtual community of practice**

- Some interview participants were unfamiliar with CAPS-CPCA, notably French-speaking physicians from Quebec. Interviewees who were aware of the virtual community of practice and had elected not to join did not perceive it as useful to them; for example, among highly experienced abortion providers those not ready to implement mifepristone abortion practice, those who felt no need for the virtual community of practice, and those who perceived that they had no time for it. A physician who had integrated mifepristone protocols and materials into their electronic medical record felt “like there’s nothing that a support group would help me with” [Physician 025].

## Discussion

### Principal Findings

In our intrinsic case study, we describe the development and use of a VCoP for mifepristone abortion providers during the first 30 months of its availability in Canada—a jurisdiction free of the legal and regulatory restraints present in many countries [15,17-19]. The alignment of findings from our website, surveys, and interviews demonstrates that CAPS-CPCA provided important support for some physicians wanting to implement this new practice. Our recruitment of 430 physician VCoP members from all regions of the country shows that many potential new medication abortion providers wanted support when mifepristone was introduced. Although we do not know what proportion of Canadian abortion providers these 430 physicians represent, this number is sizable. A 2012 study found fewer than 300 physicians across Canada who were providing abortions, most of them surgical [28]. Of our 222 CAPS-CPCA survey respondents, 140 (63.1%) had previous abortion experience, 111 (50%) had no experience with medication abortion, and 78 (35.1%) were new providers who had no experience with any type of abortion care. These findings suggest that the number of abortion providers is increasing, and emerging evidence supports this [52]. More than half (123/222, 55.4%) of the CAPS-CPCA survey respondents were from areas outside the large metropolitan centers where abortion services in Canada are concentrated [28]. New abortion providers, working in areas where services are lacking, could increase equity of access to abortion for Canadians.

Recruitment to CAPS-CPCA was very low in Quebec, and most Quebec physicians interviewed were unaware of it. System readiness for this innovation was low and inflexible in Quebec. The College of Physicians of Quebec placed explicit restrictions on the conditions for prescribing mifepristone, and physicians perceived administrative complexities to implementing medication abortion protocols. There was also a noted resistance among surgical abortion providers who did not see a relative advantage of medication abortion [25,43]. These factors slowed implementation of mifepristone abortion in Quebec, decreasing the VCoP’s utility for physicians in that province and the likelihood that they would take the Society of Obstetricians and Gynaecologists of Canada medical abortion training program whose link to the VCoP was a primary means of recruitment [43]. Another reason for Quebec’s low recruitment may relate to the province’s long-standing networking organization for abortion providers, Le Comité de vigilance sur l’avortement, which meets in person 4 to 5 times a year for discussions and education on abortion and related subjects. These abortion providers may have seen few advantages to joining the CAPS-CPCA VCoP [Edith Guilbert, personal communication].

We found that knowing that there was a VCoP for support was important for many physician members when they were considering providing medication abortion. Whether this knowledge encouraged some physicians who were interested in, but uncertain about, providing medication abortion is unknown. Our qualitative data suggested that CAPS-CPCA membership increased participants’ perceptions of confidence about providing abortion care, which is a determinant of adoption of new practices [53]. Aside from the reassurance of knowing that there was a place to access information and experts, our surveys and interviews as well as the website traffic and downloads indicated that members particularly valued the clinical practice tools. The large number of views and downloads of materials such as patient consent forms and information sheets, clinical checklists and guidelines, and members’ comments showed the site’s value as a resource repository. Although most resources were noted to be generally useful, locally relevant ones such as provincial billing codes, drug coverage, and pharmacies stocking the drug were also very important. In surveys and interviews, physician members indicated a desire for a more extensive list of pharmacies stocking the medication. Although CAPS-CPCA had more than 300 pharmacist members, fewer than 100 entered data on their pharmacy location and indicated that they had mifepristone in stock. Future VCoPs of this sort could consider approaching the large chain pharmacies for a universal input of all locations.

Logging in to the website was a deterrent for many CAPS-CPCA members, with 26.8% (34/127) of the survey respondents never doing so. To increase accessibility, we placed highly requested resources such as the clinical checklists on the landing page of the site and the very large number of views and downloads reflects the success of this strategy. Although some members found emails to be too frequent, many appreciated the emails that engaged them directly and felt that the emails built a sense of community among individuals interested in abortion. As has been shown in other research, we hypothesize that associating with other like-minded individuals may have overcome isolation and stigma that could deter some from providing abortions [29-31]. Participation in polls allowed active engagement in the VCoP to contribute data that influenced policy changes that affected members’ practice, and emails kept them apprised of these changes. Although we anticipated that member involvement would diminish over time, 59.1% (75/127) of the CAPS-CPCA members who completed the 12-month survey planned to remain in the VCoP and only 10.2% (13/127) stated that they would not continue to participate.

CAPS-CPCA provided rapid access to experts for clinical questions over the study period, with 52 *Ask an Expert* and *Discussion Room* threads related to health system, logistical, and clinical support needs. Although mifepristone abortion care is usually straightforward, we were surprised that these resources were used so infrequently. The qualitative data suggested that although this feature might be important for a few providers, most would rather use or develop their own local network for expert clinical backup. Our results from interview participants who acted as mentors suggest that over time this may have occurred, with CAPS-CPCA used as a resource to support their clinical mentorship. Nevertheless, there was a desire to have clinical support or mentorship available and this was particularly valuable for new or inexperienced providers. Although challenging to achieve, linking remote or isolated clinicians with an expert mentor in their region could provide valuable, more sustainable clinical support.

### Comparison With Prior Work

Similar to the findings of Carpenter et al [2] in their evaluation of learning communities in the United States, important elements of the CAPS-CPCA VCoP included credibility and trustworthiness achieved through affiliation with members’ professional organizations, dissociation from the pharmaceutical industry, and leadership by known experts; active and personalized outreach to engage interested clinicians; features designed to overcome known implementation barriers and share facilitators; and responsiveness to the needs of the VCoP. We responded to early requests from busy new providers to create the clinical checklists and guidelines that became CAPS-CPCA’s most viewed and downloaded resources. A qualitative study of decentralization of medication abortion services in rural Australia identified that sharing protocols and clinical resources, as we did with CAPS-CPCA, was an important enabler of clinician uptake of mifepristone abortion practice [54]. Ease of use, accessibility, and perceived usefulness have been found to be important to the success of VCoPs [2,5,55], and this was reflected in our finding that although emails and tools for practice were valued, logging in to a website was burdensome and inhibited participation for some members.

### Future Directions

Sustainability of the VCoP is uncertain; continuing usefulness for members is likely to diminish as they develop experience with mifepristone abortion and connection to local experienced mentors and experts. However, with continued diffusion of this innovation, it may continue to have relevance for new abortion providers, including nurse practitioners and, potentially, midwives. Sustainment of adoption of the innovation is an important outcome that we could not measure during our study period and an area for future research. In our interviews, lack of demand had prevented some interested physicians from implementing this practice and we hypothesize that it could similarly affect sustainment.

Our VCoP model may have applications to other clinical innovations, particularly those in focused areas where there is limited clinical experience, rapidly changing practice, unusual regulation, or associated stigma. Notably, there are established VCoPs in some jurisdictions supporting knowledge, practice, and shared experience for clinical areas such as medical assistance in dying, treatment of opioid or alcohol use disorders, and more recently COVID-19 [56-58]. Similar to CAPS-CPCA, membership in these VCoPs is not driven top-down by an organization but by individual members’ interest and motivation to deliver care in these areas. For stigmatized areas of practice such as medical assistance in dying, restricting VCoP membership to ensure that members feel safe may be important. Our VCoP also kept member identities anonymous but this may have restrained social interaction. Some CAPS-CPCA members identified this as a limitation and desired local networks for personalized interaction where clinical and service issues could be discussed. Our ability to collect real-time data from our members to inform policy makers about regulatory and policy barriers to implementation was an unusual and valuable feature that could be adapted to guide health policy changes for practice improvements in other clinical areas.

### Strengths and Limitations

Our research includes limitations. We were not able to isolate physician use of the website or *Member Announcements* and, thus, website data reflect use by all clinician VCoP members and Contraception and Abortion Research Team-Groupe de recherche sur l’avortement et la contraception staff who maintained the site. We believe that staff visits were most frequent in the early months when the site became active and may have artificially elevated page visits during this period. Survey and interview data provided the richest information about the physicians who joined CAPS-CPCA and its function for them as abortion providers. However, only 51.6% (222/430) and 29.5% (127/430) of the CAPS-CPCA physician members participated in the baseline and follow-up surveys, respectively. A smaller subset was invited for an interview. Physicians who were more involved in the surveys and interviews may not reflect the whole membership. To address these concerns, we purposefully invited interviewees to represent diverse perspectives, including physicians who did not join CAPS-CPCA. The strengths of this case study include the gathering of a large qualitative data set from physicians located in all areas of the country and the alignment of the findings from the website, surveys, and interviews.

### Conclusions

A VCoP created to address barriers and facilitators to mifepristone abortion uptake engaged physicians from across Canada and supported some to implement this innovation in abortion practice, including those who had no previous abortion experience. Creating and widely disseminating awareness of an internet-based resource that includes practical tools for implementation, timely policy and practice updates, expert advice, and social connection may be particularly beneficial for remote and isolated providers and could encourage broader dissemination of clinical innovations.

## Appendix

Multimedia Appendix 1 Canadian Abortion Providers Support-Communauté de pratique canadienne sur l’avortement website screenshots.

Multimedia Appendix 2 Baseline questionnaire.

Multimedia Appendix 3 Follow-up questionnaire administered after 12 months.

## Abbreviations

CAPS-CPCA: Canadian Abortion Providers Support-Communauté de pratique canadienne sur l’avortement
CoP: community of practice
VCoP: virtual community of practice
