# Supplementary material for: A Virtual Community of Practice to Support Physician Uptake of a Novel Abortion Practice: Mixed Methods Case Study
Source: J Med Internet Res. 2022 May 5;24(5):e34302. doi: 10.2196/34302 (PMC9121225; doi:10.2196/34302)
Supplement: Multimedia Appendix 1 [file jmir_v24i5e34302_app1.pptx]

## Slide 1
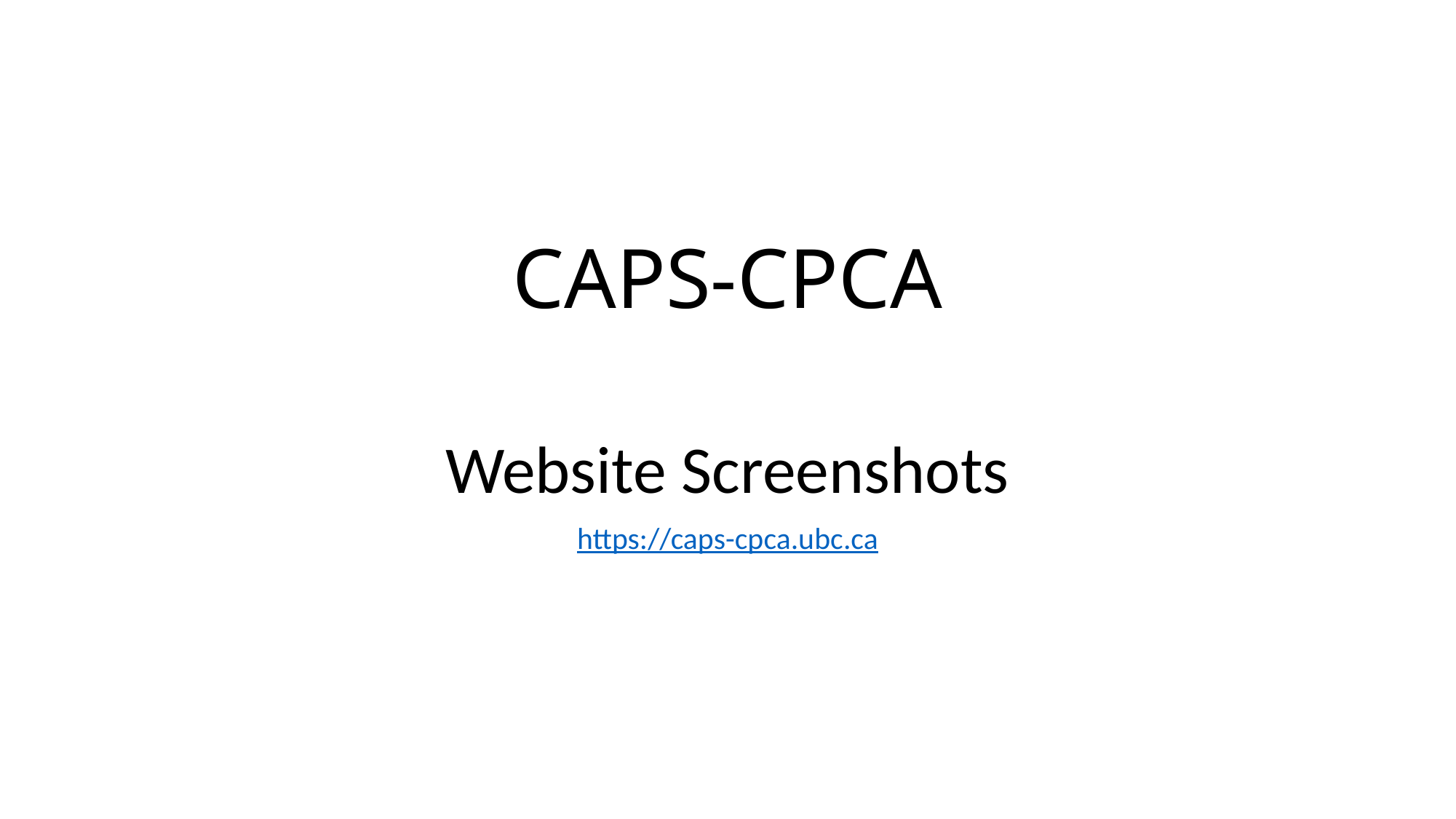

# CAPS-CPCA
Website Screenshots
https://caps-cpca.ubc.ca

## Slide 2
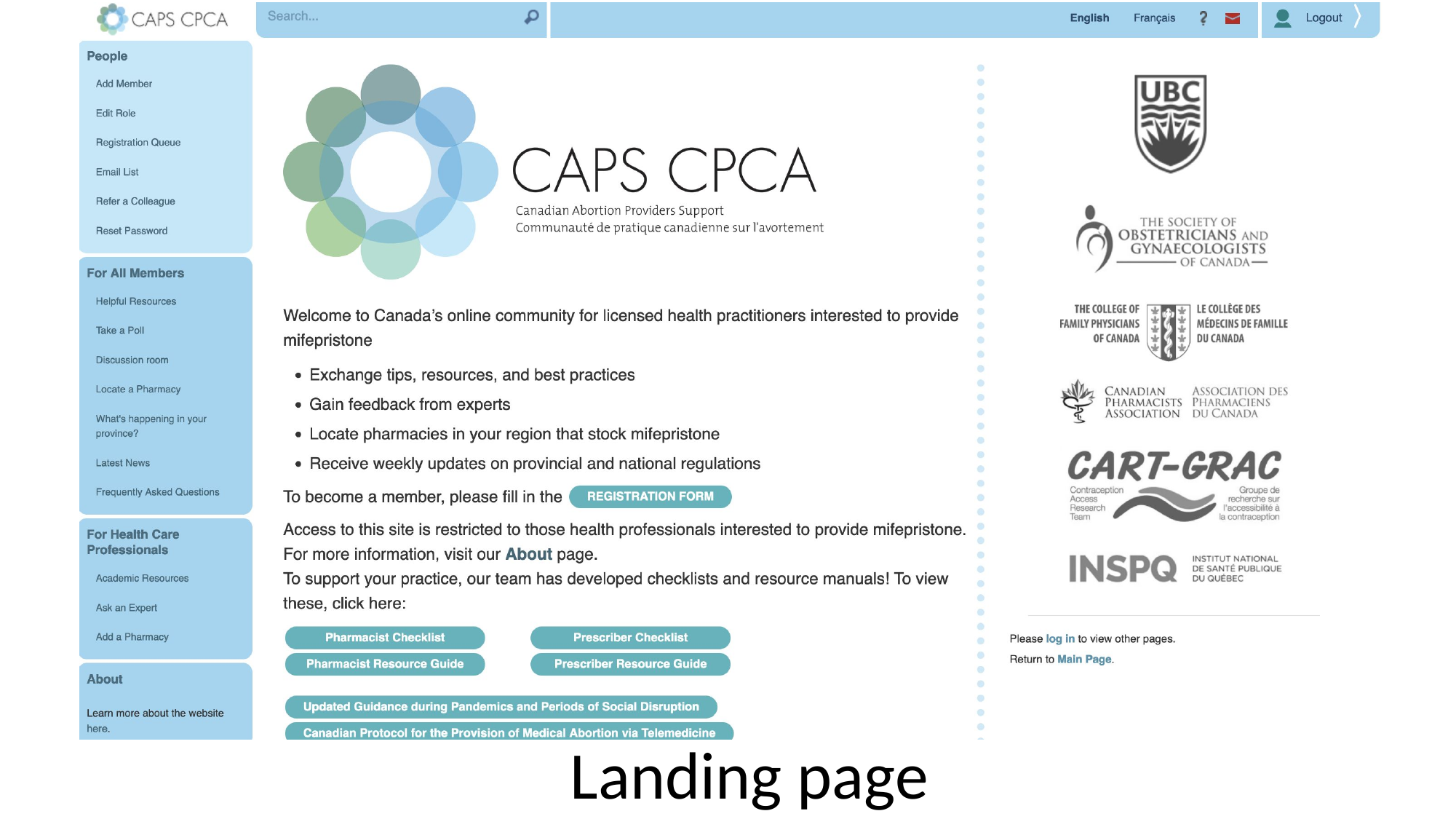

Landing page

## Slide 3
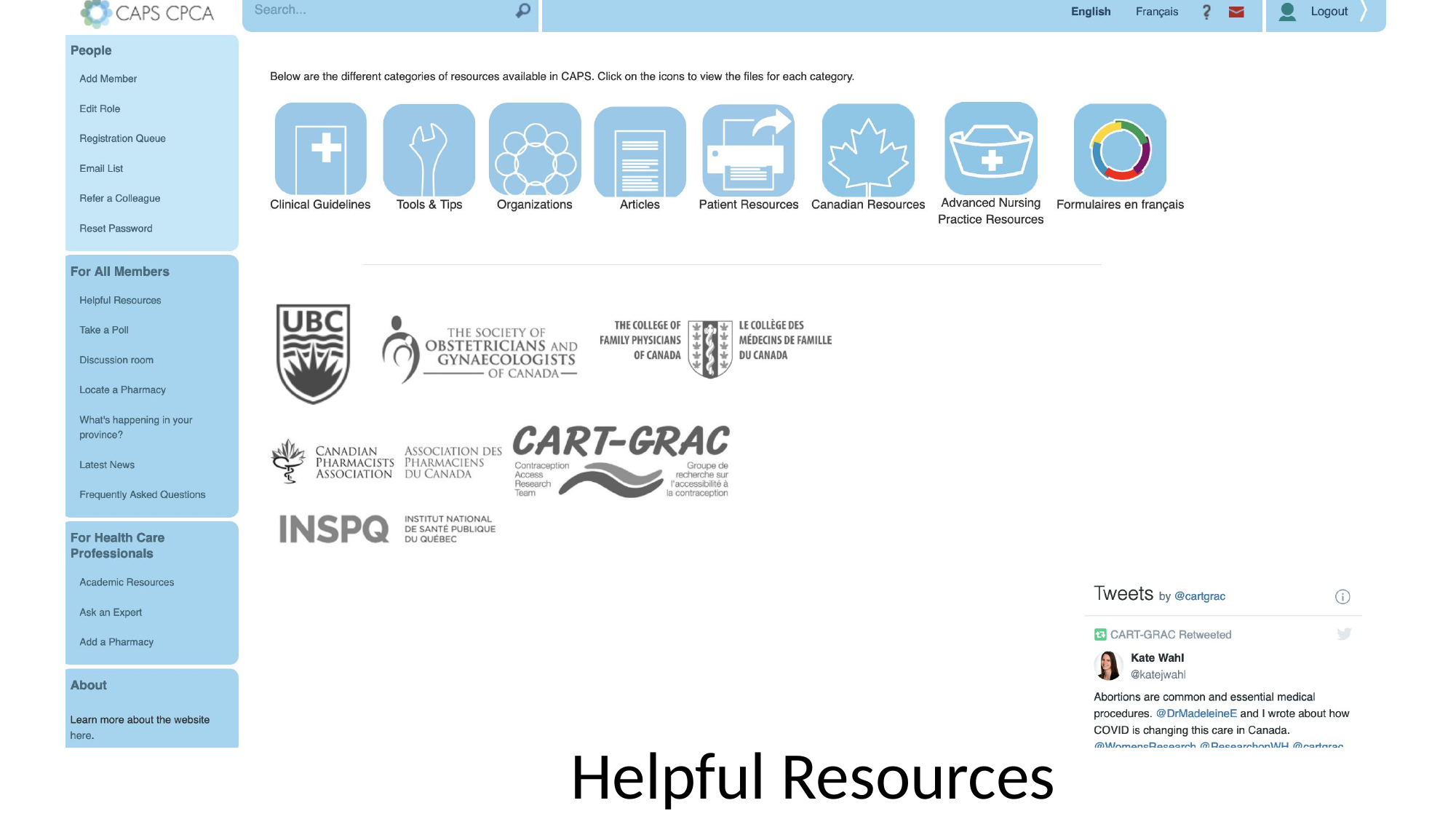

Helpful Resources

## Slide 4
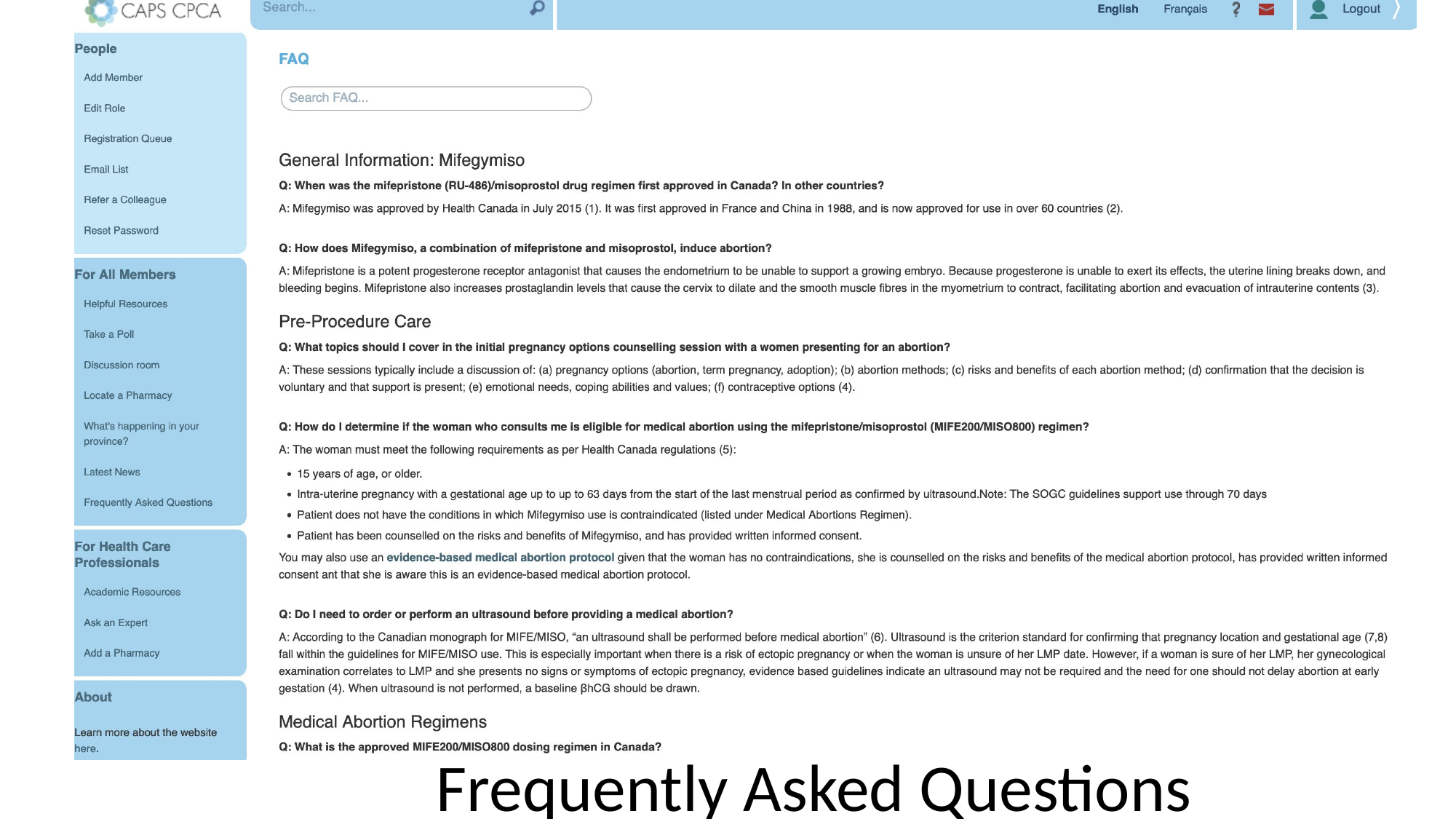

Frequently Asked Questions

## Slide 5
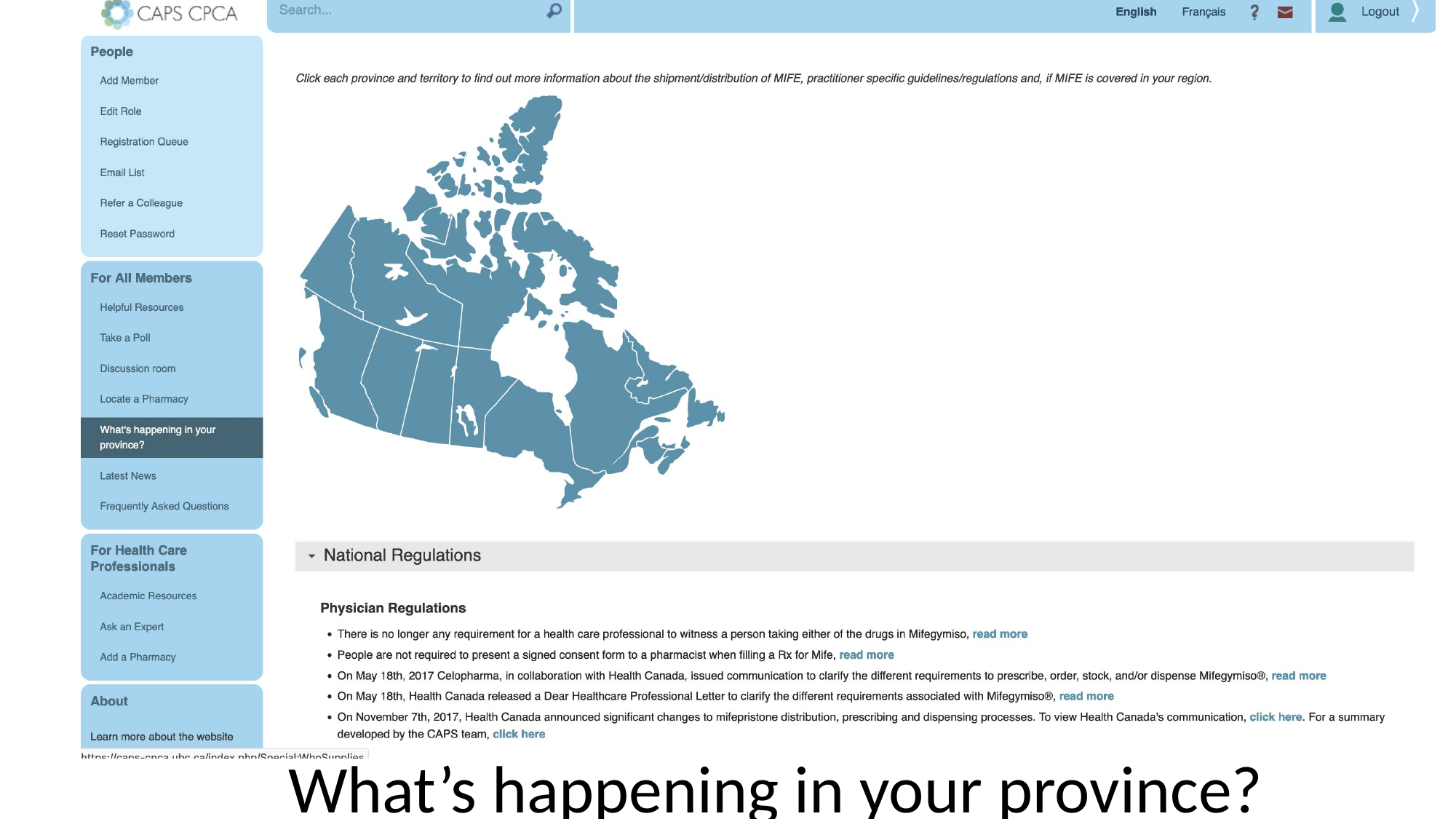

What’s happening in your province?

## Slide 6
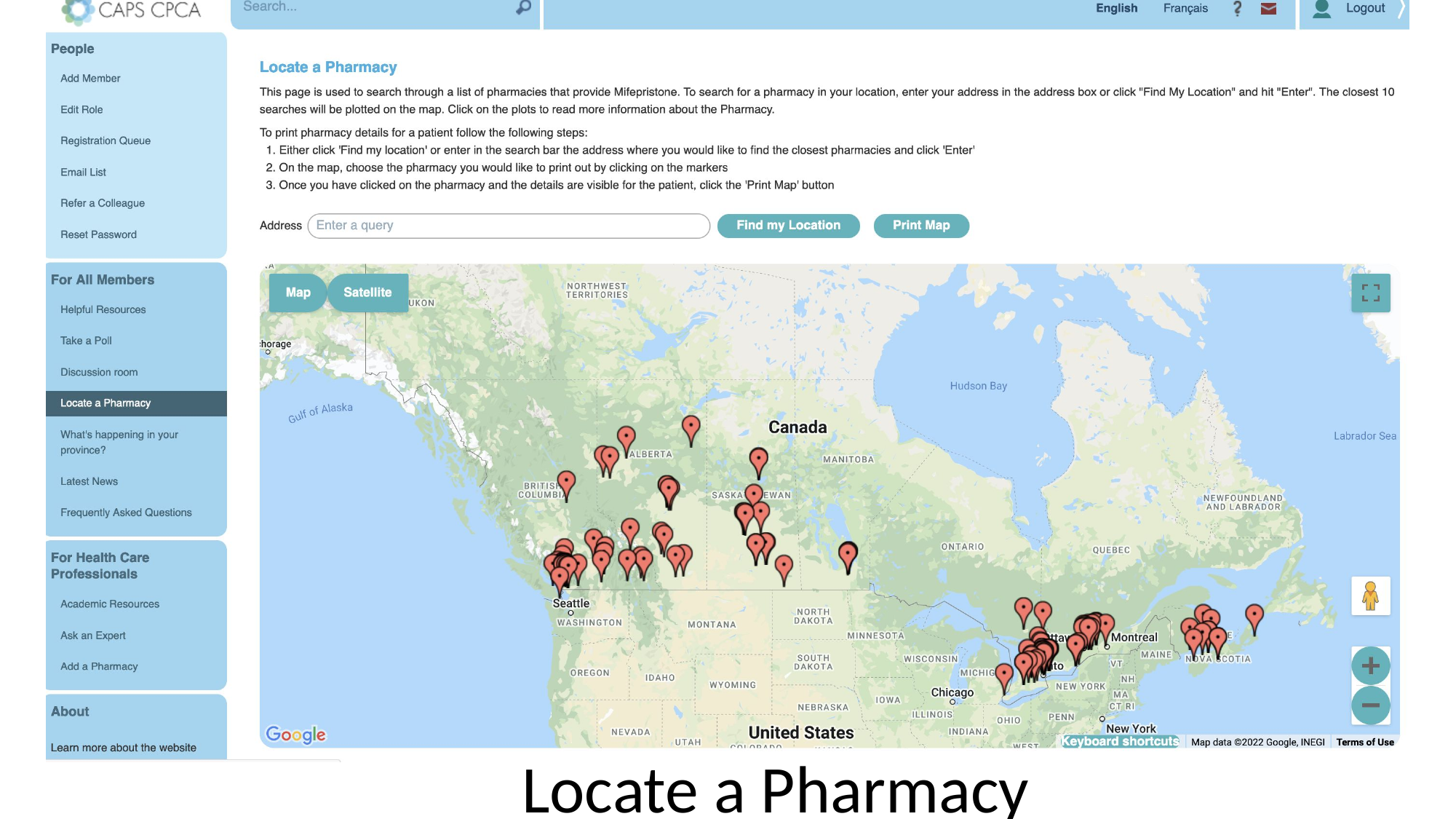

Locate a Pharmacy
