# Supplementary material for: A Virtual Community of Practice to Support Physician Uptake of a Novel Abortion Practice: Mixed Methods Case Study
Source: J Med Internet Res. 2022 May 5;24(5):e34302. doi: 10.2196/34302 (PMC9121225; doi:10.2196/34302)
Supplement: Multimedia Appendix 2 [file jmir_v24i5e34302_app2.docx]

# Mifepristone Implementation Baseline Questionnaire ^a^

1. What is your age? __________________________________
2. What is your sex?
   - Male
   - Female
   - Other
   - Prefer not to say
3. What is your primary specialty?
   - OB/GYN
   - Family Practice / Family Medicine / General Practice
   - Internal Medicine
   - Emergency Medicine
   - Paediatrics / Adolescent Medicine
   - Medical Student / Resident
   - Other

If you selected “other” above,

- - - 1. Please specify: _______________________________________________

1. Are you currently a Fellow or Certificant of the Royal College of Physicians and Surgeons of Canada, or the College of Family Physicians of Canada?
   - FRCSC or FRCPC
   - CCFP
   - None
   - Not Applicable
2. Province or Territory of your primary practice:
   - Alberta
   - British Columbia
   - Manitoba
   - New Brunswick
   - Newfoundland and Labrador
   - Nova Scotia
   - Northwest Territories
   - Nunavut
   - Ontario
   - Prince Edward Island
   - Quebec
   - Saskatchewan
   - Yukon
   - Outside of Canada
3. What is the postal code of your primary practice? __________________________________
4. University at which you completed medical school:
   - University of Alberta
   - University of Calgary
   - University of British Columbia
   - University of Manitoba
   - Memorial University of Newfoundland
   - Dalhousie University
   - McMaster University
   - Northern Ontario School of Medicine
   - Queen's University
   - University of Western Ontario
   - University of Ottawa
   - University of Toronto
   - Université Laval
   - McGill University
   - Université de Montréal
   - Université de Sherbrooke
   - University of Saskatchewan
   - International
5. University at which you completed post-graduate training: __________________________________
6. Year of completion of post-graduate training: __________________________________
7. Have you previously provided abortion services?
   - Medical abortion
   - Surgical abortion
   - Both
   - Neither

If you selected “medical abortion” or “both” above, please provide:

- - - 1. Years of experience performing medical abortions after final post-graduate training (i.e. after residency/fellowship, etc.): __________________________________

If you selected “surgical abortion” or “both” above, please provide:

- - - 1. Years of experience performing surgical abortions after final post-graduate training (i.e. after residency/fellowship, etc): __________________________________

1. How many half-days per week do you spend in clinical practice? __________________________________
2. About what percentage of your clinical work focuses on contraception and abortion? __________________________________ (%)
3. Do you plan to prescribe mifepristone for medical abortions at this time?
   - Yes
   - No
4. Do you work in more than one clinic setting?
   - Yes *(If yes, for the remaining questions in this survey, please consider the single clinic in which you anticipate providing the highest number of mifepristone medical abortions and the community in which it is located)*
   - No
5. Which of the following best describes the facility where you primarily plan to provide mifepristone medical abortion services?
   - Private physicians office
   - Abortion or reproductive-health specific community health centre or clinic
   - General health care community clinic or ambulatory health centre
   - Hospital affiliated facility
   - Other
6. If you selected “other” above, please elaborate:

___________________________________________________________________

___________________________________________________________________

The Canadian Abortion Providers Support platform (CAPS-CPCA) provides resources, "Ask an expert" rapid response, "Find-a-pharmacy" that stocks mifepristone, and a confidential communication platform for mifepristone providers.

1. Are you a registered member of CAPS-CPCA?
   - Yes
   - No

If you selected “no” above, please answer the following:

- - - 1. Do you plan to participate in this forum?
  - Yes
  - No
  - I don't know

1. Are there abortion services currently available in your community?
   - Surgical abortion
   - Medical abortion
   - Both
   - Neither
