# Supplementary material for: A Virtual Community of Practice to Support Physician Uptake of a Novel Abortion Practice: Mixed Methods Case Study
Source: J Med Internet Res. 2022 May 5;24(5):e34302. doi: 10.2196/34302 (PMC9121225; doi:10.2196/34302)
Supplement: Multimedia Appendix 3 [file jmir_v24i5e34302_app3.pdf]

Mifepristone Implementation: Physician 1-Year Follow-up Questionnaire[a]

Q#	Greenhalgh Domain	Question	Response options	Mandatory (M) Optional (O)
The survey will now commence. If you must save and return later, you can, provided you record the "return code" given to you when you leave the survey				
	**Mandatory Questions** – Are your providing mife and how many			
1	CAI 2 (& ADP 2a)	Have you provided medical abortion using mifepristone?	Yes<br>No	M
2	CAI 2a	[If yes] Approximately how many in the past 12 mos?	_______<br>(#)	M
3	*new	[If yes to CAI 2] Have you provided mifepristone medical abortion via telemedicine? (Telemedicine is defined here as *any setting where the prescriber is not directly able to physically examine the patient*)	Yes<br>No	M
4	*new	[If yes to telemedicine above] How many?	_______<br>(#)	O
5	ADP 2	[if yes to CAI2] What date did you first provide mifepristone medical abortion?	Dd/mm/yy	O
6	CAI 2b	[if no]<br>Why not?	Free text	M
7	CAI 3	Have you stopped providing mifepristone medical abortion?	Yes<br>No<br>Other	M
		[if other] Provide details:	Free text	
		[if yes] Why did you stop providing mifepristone medical abortion?	Free Text	
**Practice Support Questions**<br>Thank you for continuing our 1-year follow-up survey. We would very much like to ask you a few more questions about practice support. To continue our survey, **Click Next Page**<br>To complete the remainder of this survey at a different time, we will send you a follow-up survey code, **Click Save & Return Later**				

[a]Abbreviated from long questionnaire from the main CART-Mife study of barriers and facilitators to implementation to include only questions relevant to the Community of Practice

48	ANT 1	The CAPS platform provides resources like, “Ask an expert” rapid response, “Find-a-pharmacy” that stocks mifepristone, and a confidential communication platform for mifepristone providers:	(questions follow)	O
49	ANT 1b	Are you a registered member of CAPS-CPCA?	Yes<br>No	O
		[If N to ANT 1b] Have you heard of CAPS?	Yes I have heard of it<br>No I have never heard of it	O
		[if response to above is Yes I have heard of it] Why have you chosen to NOT join CAPS?	Free text	O
		[If Y to ANT 1b] Thinking back, from the time you decided to provide mife, how important was it for you to know that there was an online platform where you could receive support.	(5 point Likert scale Not Important to Very Important)	O
		One year later, how important has that community been?	(5 point Likert scale Not Important to Very Important)	
50	AND 1a	Do you plan to participate in this forum?	Yes<br>No<br>I don't know	O
51	ANT 1bi	How many times in the past year have you accessed the CAPS website?	______ (#)	O
52	ANT 1bii	Do you have any suggestions to improve CAPS?	Free text	O

[a]Abbreviated from long questionnaire from the main CART-Mife study of barriers and facilitators to implementation to include only questions relevant to the Community of Practice
